# Clinical impact of radiation-induced myocardial damage detected by cardiac magnetic resonance imaging and dose-volume histogram parameters of the left ventricle as prognostic factors of cardiac events after chemoradiotherapy for esophageal cancer

**DOI:** 10.1093/jrr/rrad040

**Published:** 2023-06-12

**Authors:** Rei Umezawa, Hideki Ota, Hidenobu Takagi, Noriyuki Kadoya, Yujiro Nakajima, Noriyoshi Takahashi, Takaya Yamamoto, Kei Takase, Keiichi Jingu

**Affiliations:** Department of Radiation Oncology, Tohoku University Graduate School of Medicine, 1-1, Seiryou-machi, Aobaku, Sendai, 980-8574, Japan; Department of Diagnostic Radiology, Tohoku University Graduate School of Medicine, 1-1, Seiryou-machi, Aobaku, Sendai, 980-8574, Japan; Department of Diagnostic Radiology, Tohoku University Graduate School of Medicine, 1-1, Seiryou-machi, Aobaku, Sendai, 980-8574, Japan; Department of Radiation Oncology, Tohoku University Graduate School of Medicine, 1-1, Seiryou-machi, Aobaku, Sendai, 980-8574, Japan; Department of Radiation Oncology, Tohoku University Graduate School of Medicine, 1-1, Seiryou-machi, Aobaku, Sendai, 980-8574, Japan; Department of Radiological Sciences, Komazawa University, 1-23-1, Setagayaku, Tokyo, 154-8525, Japan; Department of Radiation Oncology, Tohoku University Graduate School of Medicine, 1-1, Seiryou-machi, Aobaku, Sendai, 980-8574, Japan; Department of Radiation Oncology, Tohoku University Graduate School of Medicine, 1-1, Seiryou-machi, Aobaku, Sendai, 980-8574, Japan; Department of Diagnostic Radiology, Tohoku University Graduate School of Medicine, 1-1, Seiryou-machi, Aobaku, Sendai, 980-8574, Japan; Department of Radiation Oncology, Tohoku University Graduate School of Medicine, 1-1, Seiryou-machi, Aobaku, Sendai, 980-8574, Japan

**Keywords:** esophageal cancer, radiotherapy, magnetic resonance imaging, radiation-induced myocardial damage, cardiac events

## Abstract

This prospective study aimed to evaluate whether radiation (RT)-induced myocardial damage by cardiac magnetic resonance (CMR) imaging could be a predictor of cardiac events after chemoradiotherapy (CRT) for esophageal cancer and determine the dose-volume histogram (DVH) parameters of the left ventricle (LV) in predicting cardiac events. CMR imaging was performed before and 6 months after CRT in patients receiving definitive CRT. RT-induced myocardial damage was defined as abnormal CMR findings indicating myocardial fibrosis corresponding to an isodose line of ≥30 Gy. The cutoff values of the LV DVH parameters were calculated using the receiver operating characteristic curve based on the presence of RT-induced myocardial damage. The prognostic factors related to cardiac events of Grade 3 or higher were examined. Twenty-three patients were enrolled in the study. RT-induced myocardial damage by late gadolinium enhancement and/or an increase of 100 ms or higher in native T1 post-CRT was detected in 10 of the 23 patients. LV V45 was the best predictive factor for RT-induced myocardial damage with a cutoff value of 2.1% and an area under the curve of 0.75. The median follow-up period was 82.1 months. The 5- and 7-year cumulative incidences of cardiac events of Grade 3 or higher were 14.7 and 22.4%, respectively. RT-induced myocardial damage and LV V45 were significant risk factors (*P* = 0.015 and *P* = 0.013, respectively). RT-induced myocardial damage is a significant predictor of cardiac events. LV V45 is associated with RT-induced myocardial damage and subsequent cardiac events.

## INTRODUCTION

Chemoradiotherapy (CRT) is one of the main treatments for esophageal cancer. Some studies have demonstrated comparable treatment outcomes between CRT and surgery for esophageal cancer[1–3], and long-term survival can be expected with CRT, especially in early stage of esophageal cancer [4]. However, cardiac events, such as late toxicity after CRT for esophageal cancer, have become an important concern [5]. One of the risk factors for radiation (RT)-induced heart disease (RIHD) has been reported to be a high cumulative dose of RT (>30 Gy) [6], and some studies have reported that cardiac events were observed 7–8 months after RT for esophageal cancer [7, 8]. Therefore, early prediction of cardiac events after CRT may be important.

Various RIHDs including pericarditis, coronary disease, cardiomyopathy, heart failure, valvular disease and conduction failure after definitive RT for esophageal cancer have been reported [9, 10]. Although some studies have evaluated the frequency of RIHD using dose-volume histogram (DVH) parameters of the whole heart (WH) [7, 11], it is difficult to evaluate DVH parameters in each part of the heart, such as the heart valve and coronary artery, because of its complicated structure. Among these structures, we focused on the myocardium of the left ventricle (LV) and showed that RT-induced myocardial damage indicating myocardial fibrosis was detected by cardiac magnetic resonance (CMR) imaging, both retrospectively and prospectively [12–14]. We hypothesized that RT-induced myocardial damage detected using CMR would be associated with subsequent cardiac events. However, few studies have evaluated these aspects. This study aimed to investigate whether RT-induced myocardial damage identified by magnetic resonance imaging (MRI) at earlier periods after CRT could be a predictor of cardiac events during long-term follow-up in patients with esophageal cancer who had undergone CRT. Moreover, the relationships between LV DVH parameters and long-term cardiac events were evaluated.

## MATERIALS AND METHODS

### Study protocol and patients

This study was a subanalysis of a prospective observational study (UMIN000032551, https://upload.umin.ac.jp/cgi-open-bin/ctr_e/ctr_view.cgi?recptno=R000037126), the design and results of which have been previously reported [13]. This study was approved by the local institutional review board (2010-59), and all patients provided written informed consent before enrollment.

Patients who underwent definitive CRT for esophageal cancer between 2013 and 2015 were prospectively enrolled in the present study. The main eligibility criteria were as follows: age, 20–75 years; stage [Union for International Cancer Control (UICC) 7th], I, II, III or IV with supraclavicular or paraaortic lymph node metastasis; Eastern Cooperative Oncology Group performance status, 0–1; estimated glomerular filtration rate ≥ 30 ml/min/1.73 m^2^; no history of cardiac disease except for hypertension; patient whose myocardium was expected to be included in the RT field; patient whose long-term survival could be expected by definitive treatment; no other active malignancy at the start of our study; and no history of mediastinal radiotherapy or chemotherapy.

### CRT

The CRT and RT regimens were described in our previous report [15]. Generally, two cycles of concurrent chemotherapy (2-h infusion of cisplatin at 70 mg/m^2^ on Day 1 and continuous infusion of 5-fluorouracil at 700 mg/m^2^ for 24 h on Days 1–4) with a 4-week interval were administered during RT.

Three-dimensional conformal RT was administered to all patients. The initial clinical target volume (CTV) was defined as the region from the supraclavicular to celiac lymph nodes. Boost CTV was defined as the primary tumor with a 20- to 30-mm craniocaudal margin and an ~5-mm radial margin, and nodal metastasis. The planning target volume was defined as the CTV plus a 5- to 15-mm margin. Generally, the initial CTV dose was ~40 Gy, using parallel opposed anterior and posterior fields with or without the field-in-field technique, and the boost CTV received 20 Gy using parallel oblique fields to avoid the spinal cord. The dose distribution was determined using an ECLIPSE Varian Medical Systems (Palo Alto, CA, USA) with an analytical anisotropic algorithm.

The LV was contoured by a RT oncologist according to Feng *et al.* [16]. The inner cavity of the LV was excluded. The WH, left main artery, left anterior descending artery (LAD), left circumflex artery (LCX) and right coronary artery were also contoured. V5 (the percentage of the volume receiving 5 Gy in the LV), V10, V15, V20, V25, V30, V35, V40, V45, V50, V55, V60 and the mean dose of the LV and WH were calculated as DVH parameters. Only mean dose of the left main artery, LAD, LCX and right coronary artery was calculated because of the small volume sizes.

### CMR protocol

CMR was performed before and 6 months after CRT using the protocol described in a previous study [13, 14]. CMR imaging was performed using a 3-T whole-body scanner (MAGNETOM Trio A Tim System; Siemens Healthcare, Erlangen, Germany). Late gadolinium enhancement (LGE) imaging and T1 mapping were performed 10 min after the injection of 0.15 mmol/kg of Gd-DTPA (gadopentetate dimeglumine (Magnevist; Bayer, Osaka, Japan). LGE imaging of the LV was performed in 15 short axial sections during the diastolic phase. All T1 maps were acquired using a breath-hold-modified Look-Locker inversion recovery technique. Myocardial T1 was measured by placing the region of interest in the irradiated ventricular septum. We investigated the presence of LGE and native T1 before CRT (pre-CRT) and at 6 months after CRT (post-CRT).

### The evaluation of CMR positive findings in RT-induced myocardial damage

Since there have been only a few studies on RT-induced myocardial damage, a clear definition of RT-induced myocardial damage has not been established. Therefore, based on the results of our previous studies [13, 14], abnormal findings in CMR corresponding to regions receiving 30 Gy or more were defined as RT-induced myocardial damage. Visually identified LGE or an increased native T1 value above a reference value at post-CRT were defined as positive findings at CMR. To evaluate the reference range of change in native T1 values in the interventricular septum, the average and standard deviation (SD) at pre-CRT in all patients were calculated, and cases in which the increase in value was >2 SD at post-CRT compared to that at pre-CRT were defined as positive [17, 18]. The agreement between the RT field and CMR findings was judged by a RT oncologist and two radiologists.

### Patient management

All patients underwent regular follow-up at our outpatient clinic and were referred to the Cardiology Department if any symptoms or laboratory abnormalities were suggestive of related cardiac events. Individual atherosclerotic risk factors at baseline were managed by clinicians.

### Clinical end points

The primary endpoint was composite cardiac events of Grade 3 or higher, including acute coronary syndrome, arrhythmia, heart failure, pericarditis, valve disease and cardiomyopathy, which occurred >6 months after CRT. Individual adverse cardiac events were defined and confirmed based on the Common Terminology Criteria for Adverse Events version 4.0. with reference to a previous study [19]. The secondary endpoint of our study was to investigate overall survival (OS). The worst grade among these terms was defined as the representative grade of the late cardiac events.

### Statistical analysis

Continuous variables are presented as median values and interquartile ranges (IQRs). The average and SD values are also presented among the T1 mapping values.

The cutoff values of the LV and WH DVH parameters for predicting cardiac events were determined. The receiver operating characteristic (ROC) curve was plotted to examine the correlations between RT-induced myocardial damage by CMR and the LV DVH parameters. We examined the correlations between pericardial effusion at post-CRT and the WH DVH parameters. The cutoff of LV and WH DVH parameters was calculated based on the frequency of RT-induced myocardial damage and pericardial effusion. The optimal cutoff value on the ROC curve, which corresponds to the maximum (sensitivity + specificity −1), was calculated using the Youden index [20]. The area under the curve (AUC) was calculated to examine the prediction accuracy of each DVH parameter.

The cumulative incidence of cardiac events and OS was calculated using the Kaplan–Meier method. The cumulative incidence of cardiac events was calculated from the start of CRT to the onset of cardiac events or was censored at the last follow-up. Log-rank tests were used to examine prognostic factors related to cardiac events of Grade 3 or higher. Patient demographic characteristics at pre-CRT, brain natriuretic peptide (BNP) and pericardial effusion at post-CRT, RT-induced myocardial damage at post-CRT, LV DVH parameters and WH DVH parameters were included as potential predictors in those analyses.

All statistical tests were two-sided, and statistical significance was defined as *P* < 0.05. Statistical analyses were performed using JMP15 (SAS Institute, Cary, NC, USA).

## RESULTS

Twenty-three patients were enrolled in the study. Patient characteristics are shown in Table 1 and Supplementary Table 1 (DVH parameters of the WH and coronary artery). A CONSORT diagram is shown in Supplementary Fig. 1. Patients were treated with a median total dose of 60 Gy (50.4–66 Gy). Although only one cycle of concurrent chemotherapy was performed in three patients for various reasons, such as myelosuppression, scheduled CRT was completed in all patients. None of the patients received adjuvant chemotherapy after CRT.

**Table 1:** Patient characteristics

Factors	
Age	64 years (IQR, 59–67 years)
Sex	
Male	13
Female	10
PS	
0	19
1	4
Smoking	
Yes	16
No	7
Hypertension	
Yes	7
No	16
Angiotensin-converting enzyme inhibitor or angiotensin II receptor blocker	
Yes	5
No	18
Primary site	
Cervical-lower thoracic	1
Middle thoracic	13
Lower thoracic	9
Pathology	
Squamous cell carcinoma	23
Stage (UICC 7th)	
T1aN0M0	2
T1bN0M0	15
T2N0M0	3
T3N0M0	1
T3N1M1b	2
The cycle number cycle of concurrent chemotherapy (FP)	
One cycle	3
Two cycles	20
Pericardial effusion at post-CRT	
Yes	13
No	10
BNP at pre-CRT	14.4 pg/ml (IQR, 10.1–22.3 pg/ml)
BNP at post-CRT	35.6 pg/ml (IQR, 19.2–60.8 pg/ml)
RT-induced myocardial damage	
Yes	10
No	13
LV Mean dose	17.3 Gy (IQR, 11.8–23.4 Gy)
LV V5	53.6% (IQR, 40.0–73.0%)
LV V10	44.4% (IQR, 29.6–59.5%)
LV V15	36.9% (IQR, 26.0–51.7%)
LV V20	33.8% (IQR, 23.0–48.6%)
LV V25	30.1% (IQR, 20.4–43.4%)
LVV30	27.9% (IQR, 17.6–40.9%)
LV V35	24.0% (IQR, 14.6–38.1%)
LV V40	12.5% (IQR, 7.5–30.9%)
LV V45	2.10% (IQR, 0.0–9.20%)
LV V50	1.5% (IQR, 0.0–10.5%)
LV V55	0.6% (IQR, 0–4.10%)
LV V60	0% (IQR, 0.0–0.5%)

PS = performance status; FP = 5-fluorouracil/cisplatin.

Although pericardial effusion was detected in 13 patients 6 months after CRT, there were no symptoms at that time. On pre-CRT CMR, LGE was not found in any of the patients. The mean ± SD and median native T1 values in the interventricular septum at pre-CRT were 1184 ± 50.0 ms and 1169 ms (IQR, 1155–1195 ms), respectively. Based on these results, the 2 SD value of native T1 in the ventricular septum before CRT was 100 ms. Therefore, we defined an increased value of ≥100 ms in native T1 at post-CRT as positive for RT-induced myocardial damage. On post-CRT CMR, mean ± SD and median increased values of native T1 were 71.4 ± 43.0 ms and 69.0 ms (IQR, 31.5–106 ms), respectively. RT-induced myocardial damage was detected in 10 of 23 (43.5%) patients. The findings of both LGE and native T1 increase were positive in five patients. Only LGE was observed in four patients and only native T1 increase was observed in one patient. Although two patients did not undergo T1 mapping, we defined them as negative for RT-induced myocardial damage due to the absence of LGE. There were no significant differences in background characteristics between patients with and without RT-induced myocardial damage. The results of correlations between DVH parameters of LV and RT-induced myocardial damage and those between DVH parameters of WH and pericardial effusion post-CRT are shown in Table 2 and Supplementary Table 2. In the ROC analysis, LV 45 was the best predictive factor for RT-induced myocardial damage (AUC = 0.75). The cut-off value of LV V45 was 2.1%, and the sensitivity and specificity were 90.0 and 76.9%, respectively. The positive rates of RT-induced myocardial damage in patients above and below the cutoff value of 2.1% in LV V45 were 75.0 and 9.1%, respectively. LV V40, LV V50 and LV V55 were also useful predictive factors (AUC = 0.746, 0.729 and 0.746, respectively). There were no significant factors of the WH DVH parameter for predicting pericardial effusion after CRT.

**Table 2:** The results of RT-induced myocardial damage in DVH parameters of the LV using ROC

Parameter	Cutoff value	AUC	Sensitivity (%)	Specificity (%)
LV Mean dose	17.6 Gy	0.677	70.0	69.2
LV V5	53.60%	0.654	70.0	61.5
LV V10	36.50%	0.631	80.0	46.2
LV V15	33.30%	0.7	80.0	61.5
LV V20	31.80%	0.692	80.0	61.5
LV V25	29.50%	0.662	80.0	61.5
LV V30	25.80%	0.653	80.0	61.5
LV V35	23.40%	0.685	80.0	61.5
LV V40	12.50%	0.746	80.0	69.2
LV V45	2.10%	0.75	90.0	76.9
LV V50	1.30%	0.729	90.0	61.5
LV V55	0.80%	0.746	80.0	76.9
LV V60	2.80%	0.542	100.0	23.1

**Fig. 1:**
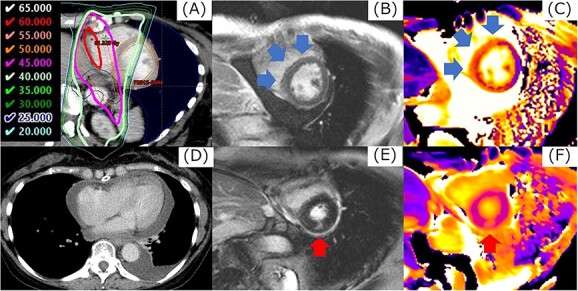
The example in cardiac event of Grade 4. Corresponding regions of 40 Gy or more (A), LGE by magnetic resonance imaging were detected in the mid-myocardial layer in the base of the LV (B). The value of native T1 in this region increased from 1168 ms before CRT to 1269 ms (C). At 53.7 months after CRT, the patient had pericardial effusion with cardiac tamponade (D). When CMR imaging was performed at the diagnosis of cardiac tamponade, a new LGE at a margin of dose distribution of 45 Gy was observed in the mid-myocardial layer in the base of the LV, separately from the LGE identified 6 months after CRT (E, F). Cardiac catheterization revealed constrictive pericarditis, and a pericardial incision was made.

**Fig. 2:**
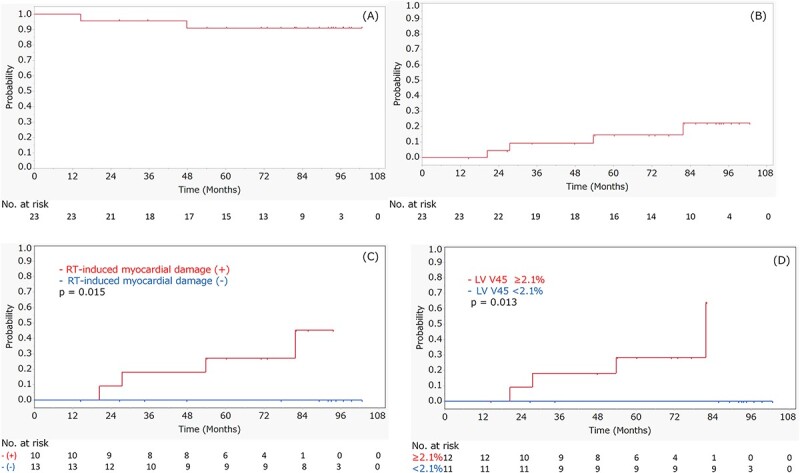
The results of OS in all patients (A), cumulative incidence of cardiac events of Grade 3 or higher in all patients (B), cardiac events of Grade 3 or higher in patients with and without RT-induced myocardial damage (C) and cardiac events of Grade 3 or higher in patients above and below the LV V45 cutoff value of 2.1% (D). Positive finding of RT-induced myocardial damage and LV V45 of 2.1% or more were significant factors to predict cardiac events.

The median follow-up period from the completion of CRT was 82.1 months (IQR: 60.2–94.5 months). Non-cardiac death was observed in two patients. One patient died of recurrent esophageal cancer 14.5 months after CRT, and the other died of bacterial pneumonia after bypass surgery for esophageal stenosis at 48 months. Cardiac events of Grade 3 or higher were observed in 4 patients at a median of 40.6 months after CRT. The details of patients with Grade 3 or higher cardiac events are shown in Table 3. An example of a Grade 4 cardiac event is shown in Fig. 1. The 7-year OS rate was 90.9% (95% confidence interval [CI], 69.9–97.7%) (Fig. 2A). The 5- and 7-year cumulative incidences of Grade 3 or higher cardiac events were 14.7 (95% CI, 4.77–37.0%) and 22.4% (95% CI, 8.31–47.9%), respectively (Fig. 2B). Table 4 and Supplementary Table 3 show the individual variables and their associations with cardiac events of Grade 3 or higher. RT-induced myocardial damage was a significant predictive factor (*P* = 0.015) for cardiac events. The 5- and 7-year cumulative incidences of Grade 3 cardiac events in patients with RT-induced myocardial damage were 30.0 (95% CI, 10.0–62.4%) and 53.3% (95% CI, 17.5–86.0%), respectively (Fig. 2C). When LGE and T1 mapping were evaluated separately, T1 mapping was a significant predictive factor for cardiac events (*P* = 0.032) (Supplementary Fig. 2). Regarding DVH parameters, LV V45 was a significant prognostic factor for cardiac events of Grade 3 or higher (*P* = 0.013). The 5- and 7-year cumulative incidences of Grade 3 or higher cardiac events in patients with LV V45 of 2.1% or more were 28.4 (95% CI, 9.36–60.4%) and 64.2% (95% CI, 16.0–94.4%), respectively (Fig. 2D). No significant correlations were observed between cardiac events and other factors.

**Table 3:** The detail of cardiac events of Grade 3 or higher after CRT

Number	Age	Sex	PS	RT dose	LV V45	RT-induced myocardial damage	The period from RT completion to cardiac event	Grade	The content of cardiac event
1	65 years	Female	0	60 Gy	6.90%	Positive	53.7 months	4	Constrictive pericarditis
2	64 years	Male	0	60 Gy	2.10%	Positive	27.5 months	3	Acute myocardial infarction
3	67 years	Male	1	60 Gy	4.10%	Positive	20.4 months	3	Congestive heart failure
4	49 years	Male	0	60 Gy	9.20%	Positive	81.8 months	3	Angina pectoris

PS = performance status.

**Table 4:** The results of univariate analysis in cumulative incidences in cardiac events of Grade 3 or higher using RT-induced myocardial damage and LV dose-volume parameters

Factors		5-year cumulative incidences in cardiac events of ≥Grade 3	*P* value
LV Mean dose	≥17.6 Gy	18.2% (95% CI, 4.58–50.7%)	0.321
	<17.6 Gy	10.0% (95% CI, 1.39–46.7%)	
LV V5	≥53.6%	19.2% (95% CI, 4.81–52.8%)	0.914
	<53.6%	10.0% (95% CI, 1.39–46.7%)	
LV V10	≥36.5%	14.3% (95% CI, 3.60–42.7%)	0.707
	<36.5%	14.3% (95% CI, 1.97–58.1%)	
LV V15	≥33.3%	16.1% (95% CI, 4.03–46.6%)	0.529
	<33.3%	12.5% (95% CI, 1.73–53.7%)	
LV V20	≥31.8%	15.4% (95% CI, 3.87–45.1%)	0.541
	<31.8%	12.5% (95% CI, 1.72–53.7%)	
LV V25	≥29.5%	16.1% (95% CI, 4.03–46.6%)	0.397
	<29.5%	12.5% (95% CI, 1.73–53.7%)	
LV V30	≥25.8%	15.4% (95% CI, 3.87–45.1%)	0.541
	<25.8%	12.5% (95% CI, 1.72–53.7%)	
LV V35	≥23.4%	16.1% (95% CI, 4.03–46.6%)	0.529
	<23.4%	12.5% (95% CI, 1.73–53.7%)	
LV V40	≥12.5%	18.2% (95% CI, 4.58–50.7%)	0.275
	<12.5%	10.0% (95% CI, 1.39–46.7%)	
LV V45	≥2.1%	28.4% (95% CI, 9.36–60.4%)	**0.013**
	<2.1%	0%	
LV V50	≥1.3%	23.1% (95% CI, 7.63–52.2%)	0.06
	<1.3%	0%	
LV V55	≥0.8%	21.3% (95% CI, 5.32–56.5%)	0.127
	<0.8%	9.09% (95% CI, 1.26–43.9%)	
LV V60	≥2.8%	0%	0.787
	<2.8%	17.8% (95% CI, 5.81–43.1%)	
RT-induced myocardial damage	Yes	30.0% (95% CI, 10.0–62.4%)	**0.015**
	No	0%	

PS = performance status; BMI = body mass index. The significant *P* values are presented in bold font.

## DISCUSSION

RIHD is an important concern in terms of quality of life and prognosis in long-term survivors of esophageal cancer. To our knowledge, this is the first study to demonstrate that noninvasive cardiac imaging testing predicts cardiac events after definitive CRT for esophageal cancer. We found (i) that RT-induced myocardial damage detected by CMR was significantly associated with higher cardiac risk in long-term follow-up and (ii) that LV DVH parameters were also associated with these adverse cardiac events. Multiple studies have demonstrated that RT-induced cardiac events are associated with lower OS [7, 19], indicating that the prevention and treatment of cancer therapy cardiotoxicity are warranted [21]. Echocardiography can be used more easily than CMR for the evaluation of cardiac toxicities, and it is recommended that echocardiography be performed routinely as a screening test for RIHD [22]. A decrease in local wall motion in the RT field was detected relatively early by echocardiography in some studies [23, 24]. However, a previous study showed that MRI was more sensitive than echocardiography for identifying reduced LV function in childhood survivors of cancer [25], and we could confirm correlations between abnormal findings and dose distribution at RT treatment planning in detail using CMR. Therefore, it may be more reasonable to conduct a careful screening examination using echocardiography in cases in which CMR at post-CRT revealed abnormal findings corresponding to the RT field. Our approach may enable oncologists and cardiologists to identify high-risk patients and improve their quality of life and life expectancy through the preventive management of cardiac events. Furthermore, because our cardiovascular disease management was limited to pre-existing risk factors, the risk stratified by our approach is the natural history of RT-induced cardiac events. In the present study, the 5- and 7-year cumulative incidences of Grade 3 or higher cardiac events were 14.7 and 22.4%, respectively. The cumulative rate of cardiac events in our study was similar to that in previous studies on CRT for lung and esophageal cancers [7, 26]. Thus, our results may be applied to the general population of patients with esophageal cancer who receive CRT, focusing on the importance of predicting cardiac events after CRT.

Although DVH parameters of the WH have often been used for predicting cardiac events, there is a possibility that the LV and coronary arteries could be similarly evaluated. Atkins *et al*. [27] reported that DVH parameters of coronary artery dose were associated with cardiac events, and we may need to pay attention to high-dose irradiation to coronary arteries. However, we considered that the abnormal findings of CMR in the present study would not be caused by the coronary arteries. It was thought that the main reasons were that the abnormal findings in the present study were included in RT field, and that the distribution of the abnormal findings might be different from ischemia due to coronary artery stenosis. Moreover, it has been reported that myocardial change due to the coronary arteries occurs several years to decades after RT [9]. From those points of view, coronary arteries might not be very useful as a predictive factor for relatively early evaluation of cardiac events. Because the LV may be easier to evaluate the DVH parameters in terms of contouring, we believe that the irradiation dose to the LV is also important. The main mechanism of RT-induced myocardial damage is microvascular injury that occurs within several months after RT [9]. Capillary swelling and progressive obstruction of the vessel lumen can lead to myocardial fibrosis [28]. There have been few reports that clearly show the dose constraint of the LV DVH parameter in CRT for esophageal cancer. In the present study, LV V45 was the best predictive factor for RT-induced myocardial damage with a cutoff value of 2.1% and an AUC of 0.75, and LV V40, LV V50 and LV V55 were also useful predictive factors. Gayed *et al*. [29] reported that most perfusion defects were detected within isodose lines of ≥45 Gy using gated myocardial perfusion imaging in RT for esophageal cancer. Although some previous studies have shown correlations between WH DVH parameters and cardiac events [11, 30], there were no significant differences between WH DVH parameters and cardiac events due to the small sample size in the present study. However, LV V45 was a significant prognostic factor for cardiac events, whereas LV V50 tended to be a significant factor. Past studies on lung cancer also reported the correlations between the LV DVH parameters and cardiac events [31, 32]. Borkenhagen *et al*. [31] reported that the volume of the ventricles receiving ≥45 Gy was associated with early cardiotoxicity. Yegya-Raman *et al*. [31] reported that multiple parameters (mean dose, V5, V30 and V50) of the LV were associated with acute coronary syndrome and congestive heart failure. Based on these results, LV DVH parameters may be useful as an index to predict the onset of RIHD. We considered that reducing the dose to the LV during RT planning for esophageal cancer would lead to minimal irradiation of the coronary arteries and heart valves.

Based on the results of ours and previous studies, it is necessary to take measures to reduce the frequency of cardiac events during CRT for esophageal cancer. First, it may be necessary to consider the total prescribed dose for esophageal cancer. Previous clinical trials have not shown sufficient effectiveness of dose escalation [33, 34]. Therefore, the total dose of RT for esophageal cancer might be reasonable, even at a standard dose of 50.4 Gy in 28 fractions. Second, it may be important to use high-precision RT to reduce the dose to the LV. Some studies have reported that the dose to the heart could be reduced by using intensity-modulated radiotherapy or proton beam therapy as a measure to reduce the dose to the WH [35, 36]. Third, it may be effective not to include prophylactic regions in the RT field. Nakatani *et al*. [37] reported the treatment outcomes of involved-field irradiation and elective node irradiation with a total dose of 60 Gy for patients with T1bN0M0 esophageal cancer. They demonstrated that the frequency of late cardiopulmonary toxicities of Grade 3 or higher was lower in the involved-field irradiation group. Yamashita *et al*. [38] also reported that patients with esophageal cancer treated with involved-field irradiation had a significantly lower risk of high-grade late toxicities than those treated with elective node irradiation. Recently, involved-field irradiation with a total dose of 60 Gy in 30 fractions for Stage I esophageal cancer has often been performed at our institution. Fourth, it has been shown that the renin–angiotensin system affects RT-induced blood vessel injury and fibrosis, and animal and human studies demonstrated that the angiotensin-converting enzyme (ACE) inhibitors were effective in preventing late toxicities including cardiopulmonary complications [39, 40]. ACE inhibitors have a role in protecting from adverse cardiac remodeling and heart failure [40] and might be one of the measures to reduce cardiac events. Although a few patients were taking ACE inhibitors because of hypertension, clinical trials on ACE inhibitors to prevent cardiac events would be desirable in the future.

The present study has some limitations. First, the number of enrolled patients in the present study was small. Therefore, multivariable analysis was not performed in the present study. A further large study is warranted to assess whether these parameters are independently predictive of future cardiac events by multivariable analysis. Second, some patients took ACE inhibitors or angiotensin II receptor blockers, which may have affected the results of our study. Third, there was no histological validation of RT-induced myocardial damage in this study. However, invasive procedures with possible significant complications should be avoided in asymptomatic patients. Fourth, there was no comparison with the nonirradiated control group. Fifth, measures to compensate for respiratory movement were not performed because radiotherapy was delivered under the condition of free breathing in the present study. Therefore, the regions receiving a high-dose irradiation were susceptible to respiratory movement. Sixth, because the definition of RT-induced myocardial damage has not been established, it is doubtful whether the definition used in the present study is valid. Recently, Groot *et al*. [41] evaluated the relationship between RT dose and myocardial fibrosis using T1 mapping after CRT for esophageal cancer. The definition of RT-induced cardiac toxicity in their study was similar to that in our study. Although we set a threshold of increased native T1 value to determine the positive findings of RT-induced myocardial damage at post-CRT T1 mapping, some past studies showed that a high range of increase in native T1 (such as increase per ≥10 ms) as a variable or transferring native T1 into a categorical variable may be associated with the development of cardiac events [42]. Moreover, we demonstrated dose-dependent RT-induced myocardial damage in previous studies. Therefore, we considered that our method for evaluation of RT-induced myocardial damage would not be vague. In the future, it is necessary to increase the sample size and analyze the clear definition of RT-induced myocardial damage.

In conclusion, we demonstrated that RT-induced myocardial damage on MRI 6 months after CRT for esophageal cancer was a significant predictor of cardiac events. LV V45 is associated with RT-induced myocardial damage and subsequent cardiac events. It is important to avoid high-dose irradiation of the LV to reduce the frequency of RIHD as much as possible.

## Supplementary Material

Supplementary_Fig_1_rrad040

Supplementary_Fig_2_rrad040

Supplementary_Table_1_rrad040

Supplementary_Table_2_rrad040

Supplementary_Table_3_rrad040

## Data Availability

The data underlying this article will be shared on reasonable request to the corresponding author.

## CLINICAL TRIAL REGISTRATION NUMBER

UMIN000032551.
