# Supplementary figures and images for: Clinical impact of radiation-induced myocardial damage detected by cardiac magnetic resonance imaging and dose-volume histogram parameters of the left ventricle as prognostic factors of cardiac events after chemoradiotherapy for esophageal cancer
Source: J Radiat Res. 2023 Jun 12;64(4):702–10. doi: 10.1093/jrr/rrad040 (PMC10354843; doi:10.1093/jrr/rrad040)

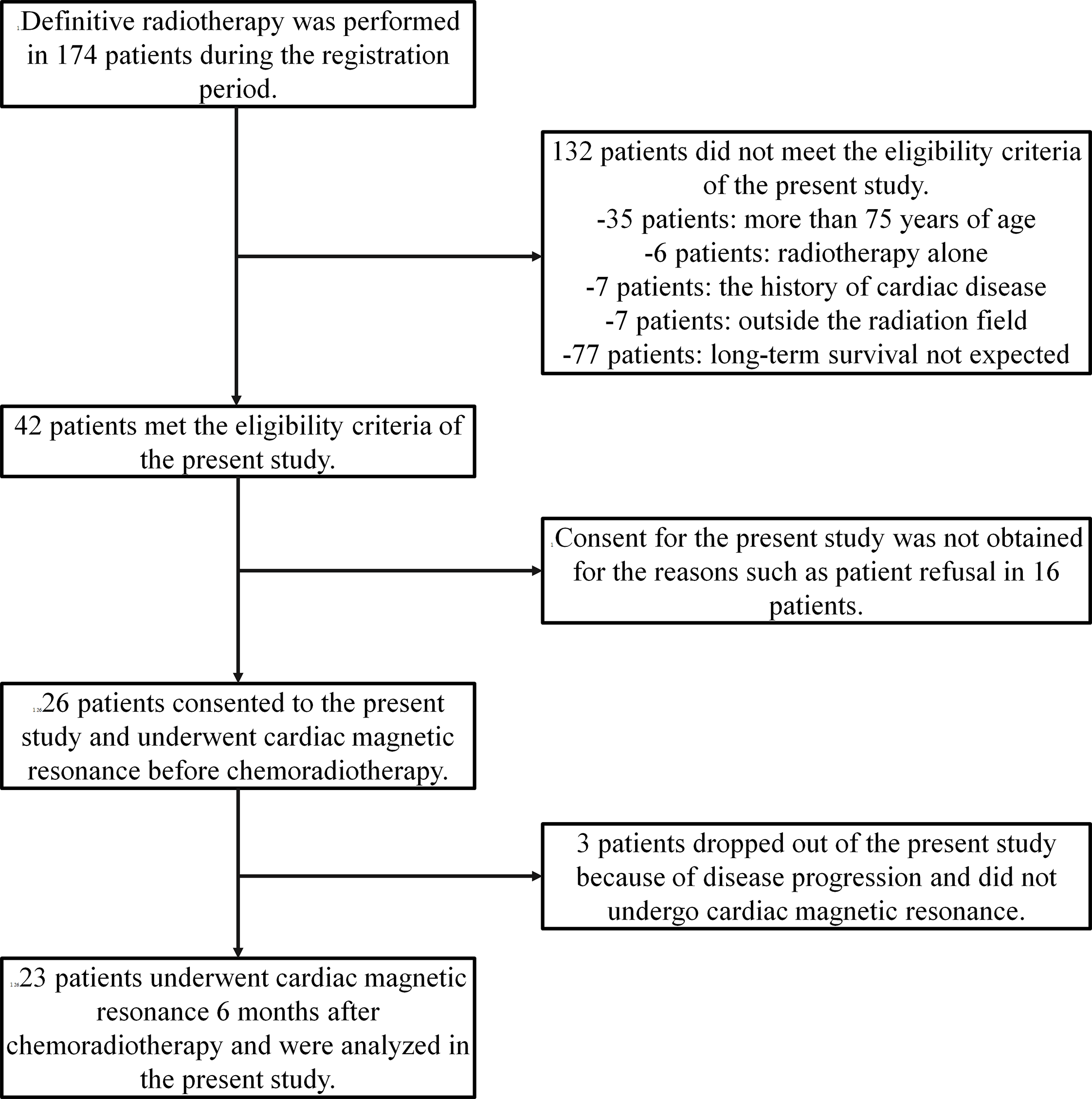

Supplement: Supplementary_Fig_1_rrad040 [file supplementary_fig_1_rrad040.zip › Supplementary_Fig_1_rrad040.tif]

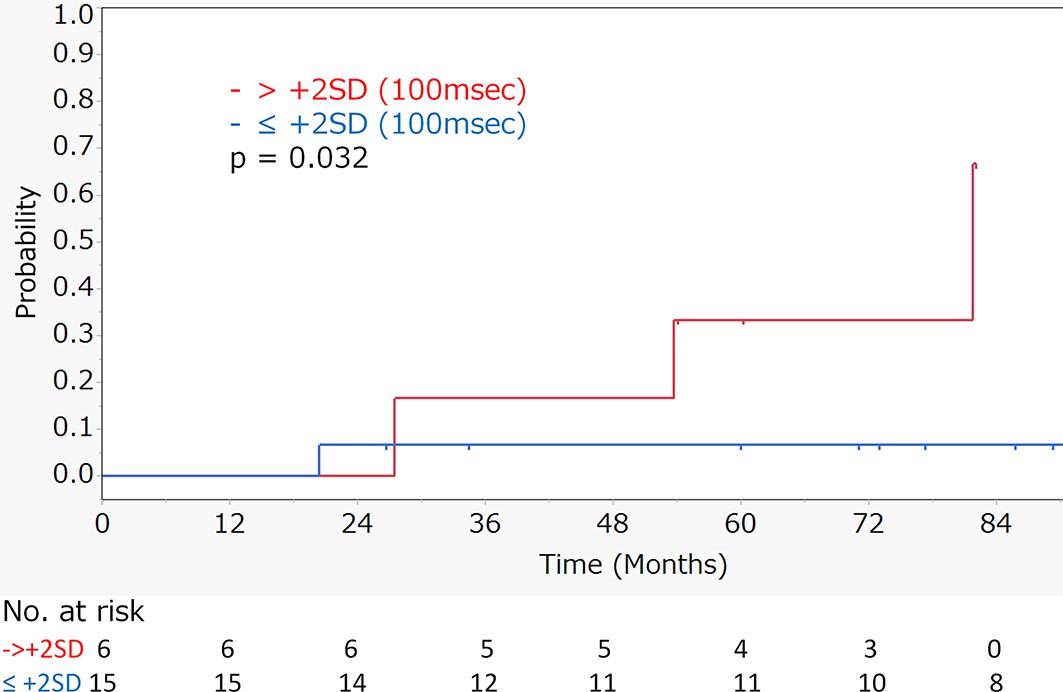

Supplement: Supplementary_Fig_2_rrad040 [file supplementary_fig_2_rrad040.jpeg]
