# Supplementary material for: Clinical impact of radiation-induced myocardial damage detected by cardiac magnetic resonance imaging and dose-volume histogram parameters of the left ventricle as prognostic factors of cardiac events after chemoradiotherapy for esophageal cancer
Source: J Radiat Res. 2023 Jun 12;64(4):702–10. doi: 10.1093/jrr/rrad040 (PMC10354843; doi:10.1093/jrr/rrad040)
Supplement: Supplementary_Table_1_rrad040 [file supplementary_table_1_rrad040.docx]

**Supplementary Table 1.** The details of dose volume parameters of whole heart and coronary arteries.

| Factor |  |
| --- | --- |
| WH Mean dose | 34.5 Gy (IQR, 30.3-38.8 Gy) |
| WH V5 | 83.2% (IQR, 78.1-92.5%) |
| WH V10 | 77.7% (IQR, 71.5-86.5%) |
| WH V15 | 73.9% (IQR, 69.6-83.0%) |
| WH V20 | 71.7% (IQR, 67.8-80.4%) |
| WH V25 | 68.4% (IQR, 63.7-73.4%) |
| WH V30 | 65.8% (IQR, 61.0-70.5%) |
| WH V35 | 63.2% (IQR, 57.8-68.5%) |
| WH V40 | 53.3% (IQR, 45.9-64.9%) |
| WH V45 | 25.9% (IQR, 13.8-33.2%) |
| WH V50 | 21.8% (IQR, 13.6-32.8%) |
| WH V55 | 13.9% (IQR, 8.10-23.8%) |
| WH V60 | 0.0% (IQR, 0.0-11.8%) |
| LM mean dose | 42.3 Gy (IQR, 39.9-44.6 Gy) |
| LCX mean dose | 30.6 Gy (IQR, 8.6-41.5 Gy) |
| LAD mean dose | 12.1 Gy (IQR, 7.1-30.1 Gy) |
| RCA mean dose | 43.0 Gy (IQR, 39.1-44.8 Gy) |

Abbreviations: WH = whole heart; heart; LM, left main coronary artery; LCX, left circumflex artery; LAD, left anterior descending artery; RCA, right coronary artery; IQR = interquartile range.
