# Supplementary material for: Clinical impact of radiation-induced myocardial damage detected by cardiac magnetic resonance imaging and dose-volume histogram parameters of the left ventricle as prognostic factors of cardiac events after chemoradiotherapy for esophageal cancer
Source: J Radiat Res. 2023 Jun 12;64(4):702–10. doi: 10.1093/jrr/rrad040 (PMC10354843; doi:10.1093/jrr/rrad040)
Supplement: Supplementary_Table_2_rrad040 [file supplementary_table_2_rrad040.docx]

**Supplementary Table 2.** The results of pericardial effusion in dose volume parameters of whole the heart using receiver operating characteristic curve.

| Parameter | Cutoff value | AUC | Sensitivity | Specificity |
| --- | --- | --- | --- | --- |
| WH Mean dose | 34.5 Gy | 0.619 | 61.5% | 60.0% |
| WH V5 | 79.20% | 0.588 | 84.6% | 40.0% |
| WH V10 | 73.00% | 0.592 | 84.6% | 40.0% |
| WH V15 | 72.30% | 0.654 | 69.2% | 60.0% |
| WH V20 | 68.30% | 0.681 | 84.6% | 60.0% |
| WH V25 | 65.00% | 0.608 | 84.6% | 50.0% |
| WH V30 | 64.00% | 0.577 | 76.9% | 50.0% |
| WH V35 | 63.20% | 0.562 | 61.5% | 60.0% |
| WH V40 | 45.90% | 0.563 | 92.3% | 40.0% |
| WH V45 | 17.70% | 0.608 | 84.6% | 50.0% |
| WH V50 | 13.60% | 0.619 | 92.3% | 40.0% |
| WH V55 | 12.00% | 0.642 | 76.9% | 60.0% |
| WH V60 | 5% | 0.654 | 61.5% | 80.0% |

Abbreviations: WH = whole heart; AUC = area under the curve.
