# Supplementary material for: Clinical impact of radiation-induced myocardial damage detected by cardiac magnetic resonance imaging and dose-volume histogram parameters of the left ventricle as prognostic factors of cardiac events after chemoradiotherapy for esophageal cancer
Source: J Radiat Res. 2023 Jun 12;64(4):702–10. doi: 10.1093/jrr/rrad040 (PMC10354843; doi:10.1093/jrr/rrad040)
Supplement: Supplementary_Table_3_rrad040 [file supplementary_table_3_rrad040.docx]

**Supplementary Table 3.** The results of univariate analysis in cumulative incidences in cardiac events of Grade 3 or higher using parameters other than radiation-induced myocardial damage and left ventricle dose volume parameters.

| Factor | | 5-year cumulative incidences in cardiac events of ≥ Grade 3 | P value |
| --- | --- | --- | --- |
| Age | ≥ 65 years | 22.2% (95%CI, 5.60-57.9%) | 0.716 |
|  | < 65 years | 8.33% (95%CI, 1.16-41.3%) |  |
| Sex | Male | 16.1% (95%CI, 4.03-39.1%) | 0.534 |
|  | Female | 12.5% (95%CI, 1.73-53.7%) |  |
| PS | 0 | 12.2% (95%CI, 3.05-37.8%) | 0.372 |
|  | 1 | 25.0% (95%CI, 3.35-76.2%) |  |
| Hypertension | Yes | 16.7% (95%CI, 2.28-63.1%) | 0.828 |
|  | No | 13.0% (95%CI, 3.25-39.7%) |  |
| Smoking | Yes | 16.7% (95%CI, 2.28-63.1%) | 0.753 |
|  | No | 14.3% (95%CI, 1.97-58.1%) |  |
| Pericardial effusion at post-CRT | Yes | 23.9% (95%CI, 7.84-53.5%) | 0.493 |
|  | No | 0% |  |
| BNP at post-CRT | ≥ 40pg/mL | 27.1% (95%CI, 6.73-65.6% | 0.483 |
|  | < 40pg/mL | 7.69% (95%CI, 1.07-39.1%) |  |
| WH Mean dose | ≥ 34.5 Gy | 9.1% (95%CI, 1.26-43.9%) | 0.801 |
|  | < 34.5 Gy | 21.3% (95%CI, 5.32-56.5%) |  |
| WH V5 | ≥ 79.2% | 12.5% (95%CI, 3.25-39.7%) | 0.229 |
|  | < 79.2% | 20.0% (95%CI, 2.72-69.1%) |  |
| WH V10 | ≥ 73.0% | 12.5% (95%CI, 3.25-39.7%) | 0.229 |
|  | < 73.0% | 16.7% (95%CI, 2.28-63.1%) |  |
| WH V15 | ≥ 72.3% | 16.1% (95%CI, 4.03-46.6%) | 0.695 |
|  | < 72.3% | 12.5% (95%CI, 1.73-53.7%) |  |
| WH V20 | ≥ 68.3% | 13.9% (95%CI, 3.47-41.8%) | 0.393 |
|  | < 68.3% | 16.7% (95%CI, 2.28-63.1%) |  |
| WH V25 | ≥ 65.0% | 13.0% (95%CI, 3.25-39.7%) | 0.974 |
|  | < 65.0% | 20.0% (95%CI, 2.72-69.1%) |  |
| WH V30 | ≥ 64.0% | 13.3% (95%CI, 3.36-40.5%) | 0.813 |
|  | < 64.0% | 16.7% (95%CI, 2.28-63.1%) |  |
| WH V35 | ≥ 63.2% | 17.5% (95%CI, 4.39-49.5%) | 0.889 |
|  | < 63.2% | 11.1% (95%CI, 1.54-50.0%) |  |
| WH V40 | ≥ 45.9% | 18.9% (95%CI, 6.19-45.2%) | 0.221 |
|  | < 45.9% | 0% |  |
| WH V45 | ≥ 17.7% | 21.7% (95%CI, 7.10-50.1%) | 0.103 |
|  | < 17.7% | 0% |  |
| WH V50 | ≥ 13.6% | 18.9% (95%CI, 6.19-45.2%) | 0.221 |
|  | < 13.6% | 0% |  |
| WH V55 | ≥ 12.0% | 23.4% (95%CI, 7.66-52.9%) | 0.062 |
|  | < 12.0% | 0% |  |
| WH V60 | ≥ 5% | 21.3% (95%CI, 5.32-56.5%) | 0.164 |
|  | < 5% | 9.1% (95%CI, 1.26-43.9%) |  |

Abbreviations: PS = performance status; BNP = brain natriuretic peptide; CRT = chemoradiotherapy; WH = whole heart; RT = radiation; CI = confidence interval.
